# Signatures of Perseveration and Heuristic-Based Directed Exploration in Two-Step Sequential Decision Task Behaviour

**DOI:** 10.5334/cpsy.101

**Published:** 2025-02-11

**Authors:** Angela Mariele Brands, David Mathar, Jan Peters

**Affiliations:** 1 Biological Psychology, Department of Psychology, University of Cologne, Germany

**Keywords:** computational psychiatry, model-based, exploration, higher-order perseveration, habits, two-step task, neurocomputational endophenotypes

## Abstract

Processes formalized in classic Reinforcement Learning (RL) theory, such as model-based (MB) control, habit formation, and exploration have proven fertile in cognitive and computational neuroscience, as well as computational psychiatry. Dysregulations in MB control and exploration and their neurocomputational underpinnings play a key role across several psychiatric disorders. Yet, computational accounts mostly study these processes in isolation. The current study extended standard hybrid models of a widely-used sequential RL-task (two-step task; TST) employed to measure MB control. We implemented and compared different computational model extensions for this task to quantify potential exploration and perseveration mechanisms. In two independent data sets spanning two different variants of the task, an extended hybrid RL model with a higher-order perseveration and heuristic-based exploration mechanism provided the best fit. While a simpler model with complex perseveration only, was equally well equipped to describe the data, we found a robust positive effect of directed exploration on choice probabilities in stage one of the task. Posterior predictive checks further showed that the extended model reproduced choice patterns present in both data sets. Results are discussed with respect to implications for computational psychiatry and the search for neurocognitive endophenotypes.

## Introduction

“When we remember we are all mad, the mysteries disappear and life stands explained.” – *Mark Twain*

Or at least it starts to make a whole lot more sense. The notion that mental health is an integral part to all of our lives and may vary over time on a continuous scale constitutes the core criticism of classic, clear-cut categories of mental disorders. This perspective is captured in more recent approaches to mental health, such as *dimensional psychiatry*. In this view, symptoms exist on a spectrum, with sub-clinical variations (e.g. of depressed mood, compulsive or avoidant behaviours etc.) present in the *healthy population* (Insel et al., 2010; Robbins et al., 2012). *Transdiagnostic* research often goes hand in hand with this dimensional view but specifically tackles the traditional symptom-based categorisation and thereby partitioning of mental disorders. High rates of comorbidity present a common issue raised with regard to the current conceptualisation and point to inherent flaws (i.e. commonly co-occurring diseases might be better understood as one shared rather than two distinct entities; Insel et al., 2010; Dalgleish et al., 2020).

Research into the basic computational processes that may go awry in the case of mental disorders provides important groundwork for these approaches (Adams et al., 2016). *Computational psychiatry* has identified several key mechanisms which likely cut across traditional diagnostic lines (Montague et al., 2012; Huys, Maia, & Frank, 2016; Insel et al., 2010; Moutoussis, Eldar, & Dolan, 2017). Such computationally derived *transdiagnostic endophenotypes* might better differentiate between mental health and disease than symptom-based conceptualisations (Robbins et al., 2012; Wise & Dolan, 2020; Yip et al., 2022; Conway & Krueger, 2021).

Reinforcement Learning (RL) theory (Sutton & Barto, 2018) has been of central importance in these efforts and extensively studied (Montague et al., 2012; Huys et al., 2021; Wise & Dolan, 2020). Several key processes have emerged as promising computational endophenotypes mapping onto (sub-) clinical variation in symptoms – *model-based* (MB) control, exploration behaviour and perseveration (Goschke 2014; Addicott et al., 2017; Kool et al., 2018).

MB control utilises a model of the world to predict action outcomes and guide behaviour accordingly. It is thought to act in concert with a simpler, *model-free* (MF) system, which selects actions based on past reinforcement (e.g. Balleine & O’Doherty, 2010; Daw et al., 2011; Daw & O’Doherty, 2014). The exploration-exploitation trade-off (Addicott et al., 2017; Sutton & Barto, 2018) refers to the process of balancing between selecting novel courses of action (exploration) and doing what has worked in the past (exploitation; Daw et al., 2006; Gershman, 2018; 2019). Here, at least two strategies have been discussed (Gershman, 2018; Wilson et al., 2014; 2021): choice randomization (random exploration), e.g. via SoftMax or epsilon-greedy choice rules (Sutton & Barto, 2018) and directed exploration, which involves the specific selection of options that maximize information gain (Wilson et al., 2021). Directed exploration shares some conceptual features with MB control, as they are both assumed to be goal-oriented and to depend on more elaborate computations (Daw & O’Doherty, 2014; Gershman & Daw, 2012; Wilson et al., 2021; for diverging views regarding the dichotomy of MB & MF control see e.g. Akam et al., 2015; Doody et al., 2022; Miller et al., 2019). However, simpler heuristic-based exploration strategies may serve as computationally less costly alternatives (Fox et al., 2020). Instead of a precise model of environmental dynamics (see e.g. Kalman Filter models;. Chakroun et al., 2020; Daw et al., 2006; Speekenbrink, 2022), an agent may utilize a simple proxy measure of environmental uncertainty (and therefore of potential information gain). Perseveration, on the other hand, refers to a general tendency for action repetition (or choice stickiness). It is related to both MB control and exploration, as it is by definition linked to reduced exploration and is often thought to be associated with reduced MB control (see e.g. Voon et al., 2015). In a range of computational models, perseveration is modelled as a subjects’ propensity to repeat the directly preceding (t–1) action (*first order perseveration*; FOP). Perseveration can also be conceptualised to extend over several trials (Lau & Glimcher, 2005; Gershman 2020; Miller et al., 2019). In *Higher Order Perseveration* (HOP) subjects’ previous actions beyond the last trial (t–1) continue to exert an influence on current actions (see e.g. Bornstein & Banavar, 2023; Miller et al., 2019). Notably, these effects are independent of value (Miller et al., 2019), i.e. independent of the MF system, and may be more closely related to real-world habitual behaviour than FOP (which in turn is assumed to depict basic motoric components of habitual responding; see e.g. Bornstein & Banavar, 2023; Gershman, 2020; Miller et al., 2019).

Alterations in directed exploration and MB control are thought to underlie habitual and/or compulsive behaviours characteristic of several mental disorders (e.g. Voon et al., 2015) spanning schizophrenia (Culberth et al., 2016), substance use disorders (e.g. Addicott et al., 2013; Morris et al., 2016; Reiter et al., 2016; Sebold et al., 2017; Smith et al., 2020), pathological gambling (e.g. Bruder et al., 2021; Wiehler, Chakroun, & Peters., 2021; Wyckmans et al., 2019), eating disorders (Foerde et al., 2021; Reiter et al., 2017), and obsessive-compulsive disorder (OCD; Banca et al., 2023; Brown et al., 2020; Gillan et al., 2011; Gillan & Robbins, 2014). Similar effects were observed for sub-clinical variations in symptom severity (Gillan et al., 2016; Seow et al., 2021). Reduced MB control could thus constitute a promising transdiagnostic endophenotype that might more closely relate to real world behaviour than traditional clinical categorizations (Ferrante & Gordon, 2021; Maia & Frank, 2011). Notably, however, maladaptive behaviours in these groups have also been linked to increased perseveration (SUD: e.g. Leeman & Potenza, 2012; schizophrenia: Waford & Lewine, 2010; OCD: Banca et al., 2023; depression and eating disorders: Voon et al., 2015; Waller et al., 2012). Dysregulations in MB control, exploration and perseveration might therefore constitute potential transdiagnostic endophenotypes in computational psychiatry (Addicott et al., 2017). Interestingly, despite the fact that both MB control and directed exploration have been conceptually linked to the goal-directed system, and perseveration to downregulation thereof, they have largely been studied in isolation.

**Figure 1:**
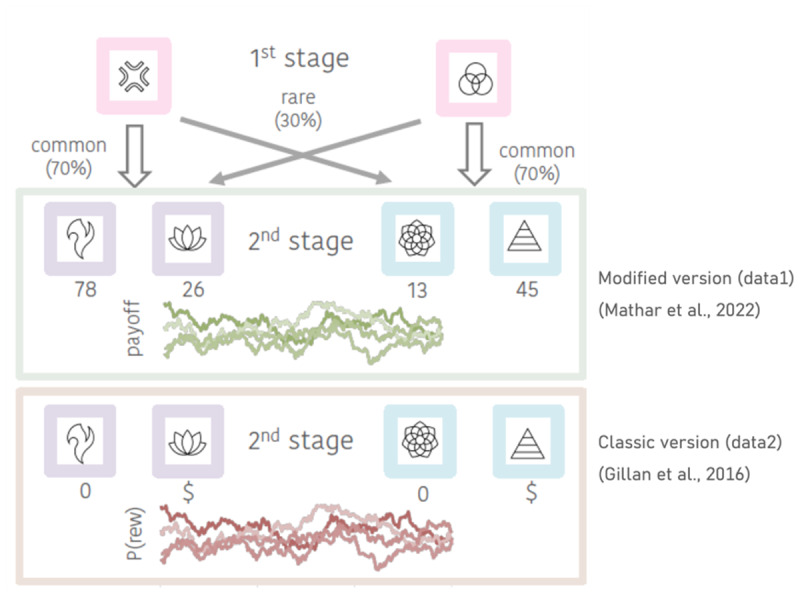
Outline of the two-step task (TST). Transition probabilities from the first stage to the second stage remain the same in both versions of the task. The second stage with a green frame depicts the modified task version employed in data set *data1* (Mathar et al., 2022): after making a S2-choice subjects receive feedback in the form of continuous reward magnitudes (rounded to the next integer). The lower S2 stage (orange frame) depicts the classic version (used in data set *data2*; Gillan et al., 2016), in which the S2 feedback is presented in a binary fashion (rewarded vs. unrewarded based on fluctuating reward probabilities).

For more than a decade, the *two-step task* (TST, Daw et al., 2011) has been one key paradigm in the study MB and MF contributions to behaviour, and a central instrument in computational psychiatry (e.g. Voon et al., 2017; Patzelt et al., 2019; Dolan & Dayan, 2013). The TST consists of two stages, which each involve a binary choice and are linked in a (stable/fixed) probabilistic manner (Daw et al., 2011; Figure 1). First-stage choices lead to one of two different second stages (S2, Figure 1) with either high (common transition, 70%) or low probability (rare transition, 30%). Second stage options are then associated with either drifting reward probabilities (*classic version*, see e.g. Daw et al., 2011; Gillan et al., 2016) or drifting reward magnitudes (*modified version*, see. Mathar et al., 2022).

Importantly, MB and MF control make different predictions for S1 choices as a function of reward and transition on the previous trial (c.f. e.g. Daw et al., 2011; Otto et al., 2013b). In this way, the TST is thought to allow for an estimation of the relative contribution of each system (Daw et al., 2011; Otto et al., 2013a).

Interestingly, the TST shares several key features with tasks traditionally used to study exploration, such as restless bandit tasks (Daw et al., 2006; Sutton & Barto, 2018; Speekenbrink, 2022; Chakroun et al., 2020; Wiehler et al., 2021). Fluctuating rewards (or reward probabilities, depending on task version) in S2 of the TST afford continuous tracking and introduce potential information gain in S2 as a function of sampling recency of these options. Given the structural similarities between S2 of the TST and restless bandit problems, it thus seems natural to hypothesize that similar directed exploration processes might contribute to TST behaviour. One way to investigate this possibility is to specify and implement proposed additional processes within computational models, which can subsequently be tested with regard to their fit to empirical data.

The present study followed this approach. Our primary aim was an extension of standard hybrid models of MB and MF control for the TST by incorporating directed exploration strategies. We implemented and compared several potential candidate mechanisms, including more elaborate mechanisms of uncertainty tracking (Kalman, 1960; Daw et al., 2006) as well as simpler heuristics (Fox et al., 2020). Second, given the conceptual links between perseveration and exploration (see above), we then expanded our model space to additionally examine the role of first-order vs. higher-order perseveration on the TST. We tested and compared our models in two independent data sets, a variant of the TST with drifting reward magnitudes in S2 (*data1*, Mathar et al., 2022) as well as the classical TST with drifting reward probabilities (and binary payouts) in S2 (*data2*, Gillan et al., 2016). A wealth of empirical data from the TST have already been acquired, many of which (including data from clinical groups) are available for re-analysis. Therefore, investigations into additional computational mechanisms that might be reflected in these data could proof valuable for the field of computational psychiatry as well as mathematical psychology in general.

## Methods

### Participants and Task Versions

We evaluated all models on the basis of a re-analysis of two independent existing data sets. The first data set (*data1*) encompasses data from 39 healthy, male participants (aged 18–35; *M* = 25.17, *SD* = 3.89) who performed 300 trials of the modified TST version (neutral condition from Mathar et al., 2022). The second data set (*data2*) constitutes a subsample (N = 100) from a previously published large scale online study using the classical TST (Gillan et al., 2016).

#### Data1

The first data set (data1) was obtained in a recent study (Mathar et al., 2022) that spanned two testing sessions and included additional tasks, self-report measures and physiological markers of autonomic arousal, which were not analysed for the current study (for more details see Mathar et al., 2022). Prior to performing the TST, participants received instructions regarding the transition probabilities as well as fluctuating reward structure and performed 20 training trials. In addition, they were informed that the maximum response time was two seconds and that they could obtain an additional 4€ in reimbursement, contingent upon task performance. The original study covered two conditions, one including an experimental manipulation in the form of erotic pictures which were presented in a block-wise fashion prior to task execution and rated by the participants with regard to their valence and arousal. Data analysed here all stem from the neutral condition, which followed the same procedure, however, only contained neutral images.

This TST version used transition probabilities fixed to 70% and 30% for common and rare transitions, respectively and the reward magnitudes for each S2 option followed independent Gaussian Random Walks (fluctuating between 0 and 100, rounded to the next integer, see Fig. 1). S2 states that were marked by different colours to make them more easily distinguishable. For all analyses, trials with response times <150 ms were excluded.

#### Data2

Gillan and colleagues (2016) used the original variant of the TST (Daw et al., 2011). Here, reward probabilities of all choice options varied independently according to Gaussian Random Walks, and participants received binary reward feedback (reward vs. no reward; Figure 1). Detailed descriptions of the whole sample, exclusion criteria, procedure, additional measures, as well as specifics of the TST version employed can be found in the original publication.

We drew a subsample of N = 100 (age: *M* = 34; *SD* = 11; 69% female) from the full sample of Experiment 1 from the original publication (N = 548, age: M = 35; SD = 11; 65% female; for further details see Gillan et al., 2016). This subsample is representative of the whole original sample regarding the self-report measures obtained by the authors. To yield this subsample, we randomly sampled from the original full sample from Experiment 1 until self-reported symptom severity did not significantly differ from those of the full sample. The resulting transdiagnostic symptom-score relating to compulsivity and intrusive thoughts (c.f. PCA analyses reported in Gillan et al., 2016 for more detail) was chosen as a criterion due to its significant association with model-based RL in the original publication.

### Model-Agnostic Analyses

As a first step we used logistic mixed effects regression models to analyse *stay-probabilities* for first-stage choices, i.e. the probability to repeat the first stage selection of the preceding trial, depending on the transition (common vs. rare) and reward (rewarded vs. unrewarded) experienced. Such regression models are amongst the most common ways of analysing TST behaviour outside a computational modelling framework. Reward and transition type (rewarded/unrewarded and common/rare were coded as 1/–1 respectively) were entered as (fixed effects) predictors of S1 choice repetition (i.e. perseveration). Individual subjects were entered as random effects. Using the *lme4* package (Bates et al., 2015) this resulted in the following model specification in the R syntax:


\[
\text{pstay}\ \sim \ \text{rew}\ \text{*}\ \text{trans}\ +\ \left(\text{rew}\ \text{*}\ \text{trans}\ +\ \text{1}\ |\ \text{subj}\right)
\]

Due to the presentation of continuous reward magnitudes in the modified version (data1) we defined outcomes to be *rewarded/unrewarded* (1/–1) relative to the mean outcome over the preceding 20 trials (Wagner et al., 2022; Mathar et al., 2022).

As additional model-agnostic indices of MB and MF behaviour, we calculated difference scores (*MB_diff_ & MF_diff_*) as proposed by Eppinger and colleagues (2013):


\[
\begin{array}{c} M{{F}_{diff}}\ =\ \left[{{P}_{common}}_{,\ rewarded}\ +\ {{P}_{rare}}_{,\ rewarded}\right]-\left[{{P}_{common}}_{,\ unrewarded}\ +\ {{P}_{rare}}_{,\ unrewarded}\right] \\ \ \text{and}\ M{{B}_{diff}}\ =\ \left[{{P}_{common}}_{,\ rewarded}\ +\ {{P}_{rare}}_{,\ unrewarded}\right]-\left[{{P}_{common}}_{,\ unrewarded}\ +\ {{P}_{rare}}_{,\ rewarded}\right]. \\\end{array}\]

### Computational Models

Our final model space consisted of six models in total. Two standard hybrid RL models without exploration terms but with different accounts of perseveration: a common implementation of first-order perseveration (FOP; i.e. repetition of the preceding action) and more temporally extended higher-order perseveration (HOP; c.f. Q + FOP & Q + HOP, respectively). We extended both models with exploration terms that incorporated different ways in which S2 uncertainty might impact first stage choice probabilities (see below). In addition to the model space presented here, we also tested a variety of other alternatives. These included parallel models with a Kalman-Filter learning rule (*Bayesian Learner*) based on computational models regularly applied in the analysis of data from explore-exploit-paradigms (see e.g. Daw et al., 2006; Chakroun et al., 2020). All of these models however, provided a noticeably inferior fit to the data at hand and are thus, not described in more detail here. Further information on the pre-selection process for the final model variants can be found in the Model Comparison section below. Descriptions of the BL model variants are provided in the supplement (c.f. Section Bayesian Learner Models).

#### Learning Rule

##### Q-Learner

The learning mechanism and basis for all final model variants is an adaptation of standard hybrid models (e.g. Daw et al., 2011; Otto et al., 2013b). Here, for each S1 state-action pair (i.e. choice option), separate MF and MB values (*Q_MF_, Q_MB_*) are calculated in parallel.

MF values in both stages are updated using the TD learning algorithm SARSA (Rummery & Niranjan, 1994), such that MF Q-values of a chosen state-action pair at stage *i* on trial *t* are updated according to:


1 \[
{Q}_{MF} \left({s}_{i,t},\ {a}_{i,t}\right)\ =\ Q_{MF} \left({s}_{i,t},\ {a}_{i,t} \right)\ +\ {\alpha}_{i} {\delta}_{i,t}\ \text{with}\ \text{the}\ \text{RPE}:
\]

2 \[
{\delta}_{i,t}\ =\ {r}_{i,t}\ +\ {Q}_{MF}\left({s}_{i+1,t},\ {a}_{i+1, t}\right)\ -\ {Q}_{MF} \left({s}_{i,t},\ {a}_{i,t}\right).
\]

and a constant learning rat *α_i_* (ranging from 0 to 1) for each stage.

S2 prediction errors are incorporated into S1 value estimates via the second-stage learning rate *α*_2_ (constrained between 0 and 1):


3 \[
{Q}_{MF} \left({s}_{1, t}, {a}_{1, t}\right)\ =\ {Q}_{MF} \left({s}_{1, t}, {a}_{1,t}\right)\ +\ {\alpha}_{2} {\delta}_{2,t}.
\]

While several other models utilize an *eligibility trace* parameter (*λ*) to propagate S2 RPEs, here we chose to reduce the parameter space and model complexity by instead using the S2 learning rate.

We included an additional “forgetting process” for MF Q-values (Toyama et al., 2017; Toyama et al., 2019), such that unchosen Q-values decayed towards the mean according to a decay rate *α*_3_ (constrained between 0 and 1):


4 \[
Q_{MF}\left({s}_{1,t}, \bar{a}_{1,t}\right) = {\alpha}_{3} * {Q}_{MF} \left({s}_{1,t}, \bar{a}_{1,t}\right) + \left(1 - \alpha_{3}\right)\ \text{*}\ \text{0}\text{.5}\ \text{and likewise},\ \text{for S2 MF values}:
\]

5 \[
{Q}_{2}\left({{s}_{2,t}},\ {{\bar{a}}_{2,t}}\right)\ =\ {\alpha}_{3}\ *\ {{Q}_{2}}\left({{s}_{2,t}},{{\bar{a}}_{2,t}}\right)\ +\ \left(1 - {{\alpha}_{3}}\right)\ *\ 0.5.
\]

Recall that transition probabilities in the model were fixed as follows:


6 \[
\begin{array}{c} P\left({{s}_{B}} \vert {s}_{A}, {a}_{A}\right) = 0.7,\ P\left({s}_{C} \vert {s}_{A},\ {a}_{B}\right) = 0.7,\ \text{or in the alternative case as}: \\ P\left({s}_{B} \vert {s}_{A}, {a}_{A}\right) = 0.3,\ P\left({s}_{C} \vert {s}_{A}, {a}_{B}\right) = 0.3. \end{array}
\]

with


7 \[
P\left({s}_{B} \vert {s}_{A}, {a}_{B}\right) = 1 - P\left({s}_{B} \vert {s}_{A}, {a}_{A}\right)\ \text{and}\ P\left({s}_{C} \vert {s}_{A}, {a}_{A}\right) = 1 - P\left({s}_{C} \vert {s}_{A}, {a}_{B} \right)
\]

First-stage *Q_MB_* values were then computed as the maximal *Q*_2_ values weighted by their respective transition probabilities. Thus, using the Bellman equation *Q_MB_* values are defined as:


8 \[
\begin{array}{l}
{Q}_{MB} \left({s}_{A}, {a}_{j}\right) = P\left({s}_{B} \vert {s}_{A}, {a}_{j}\right) \displaystyle\mathop{\max}_{a\in \{a_{A},a _{B}\}} Q_{2} (s_{B}, a)\\
\qquad\qquad\qquad\ + P\left({s}_{C} \vert {s}_{A}, {a}_{j}\right) \displaystyle\mathop{\max}_{a\in \{a_{A},a _{B}\}} Q_{2} (s_{C}, a)
\end{array}
\]

As a trial ends with the second-stage choice, for S2 only MF values are relevant, such that:


9 \[
{{Q}_{MB}}\left({{s}_{2,t}},a\right)\ =\ {{Q}_{MF}}\left({{s}_{2,t}},\ a\right)\ =\ {{Q}_{2}}\left({{s}_{2,t}},a\right).
\]

Accordingly, *Q*_2_ (*s_2,t_, a*) updates follow the TD process as described previously for first stage *Q_MF_* values (Equation 1), while allowing for a separate learning rate *α*_2_ (also constrained between 0 and 1).

##### Exploration Bonus

Next to these classic value computations, our extended models also assume a learning and updating process for the informational value of choice options as indicated by the uncertainty associated with them. The following sections describe the different implementations of directed exploration for first stage choices in more detail. The general idea is that participants may seek out uncertain S2 states for information-gain and potential long-term reward maximization. Random exploration, in contrast, is assumed to result from sub-optimal, random deviations from a reward-maximizing decision-scheme (Sutton & Barto, 2018; Wilson et al., 2014; 2021).

We compared two different formalizations of uncertainty that participants might draw upon during directed exploration for S1 decisions (see below). These different types of exploration bonus incorporated transition probabilities analogously to the *Q_MB_* values (c.f. Eq. 8). This formalization was based on previous research efforts defining directed exploration as a goal-directed strategy, aiming at long-term reward accumulation via maximal information gain (Wilson et al., 2014; 2021). In this way directed exploration and MB control show a large conceptual overlap (i.e. deliberate forward-planning under consideration of environmental dynamics, with a long-term perspective on goal-attainment). Consequently, as formalized in the MB component, transition probabilities are utilized to weigh the uncertainty estimates (vs reward estimates for MB values) associated with S2 options to reflect these assumed deliberate and foresighted aspects.

##### Uncertainty estimates based on a bandit-counter heuristic

For the first of these (*b_n, t_*(*s_B_, a*), *bandit-heuristic*), participants were assumed to estimate how many of the alternative S2 options they have sampled since last choosing a given option *n*. Following selection of an S2 option *n*, the respective counter *b_n,t_* is reset to 0. Thus, *b_n,t_* of a given S2 option *n* ranges from 0 (this option was chosen on the last trial) to 3 (all other S2 option were sampled since last sampling this option). In line with the basic idea of a goal-directed exploration mechanism, which however, relies on a simplifies, efficient uncertainty estimate (i.e. counter-heuristic vs. full Bayesian uncertainty tracking) we assume participants to sum up these counters over both options of the respective S2 stage associated most likely with either first-stage choice (instead of tracking the maximum). The sum of *b_n,t_* across associated S2 options were again weighted by their transition probabilities (c.f. Equations 6–8) resulting in:


10 \[
\begin{array}{l} e{{b}_{bandit}}\left({s}_{A}, {a}_{j}\right)\ = & P\left({{s}_{B}}\ \vert\ {{s}_{A}},{{a}_{j}}\right)\ {\sum}_{a}{{b}_{n,t}}\left({{s}_{B}},\ a\right) \\ & +P\left({{s}_{C}}\ \vert\ {{s}_{A}},{{a}_{j}}\right)\underset{a}{\sum}\,{{b}_{n,t}}\left({{s}_{C}},\ a\right). \end{array}
\]

This yielded the exploration bonus implemented in variants *QL + BANDIT* and *QL + BANDIT + HOP* (see Equations 16 and 17 for corresponding S1 choice probabilities). Often times uncertainty sum-scores are associated with less goal-directed exploration strategies (i.e. random exploration based on total uncertainty), such as Thompson Sampling. In those cases however, total uncertainty scores (sums) are directly linked to choice stochasticity (c.f. Gershman, 2018; 2019; Fox et al., 2020). In contrast, here the summed uncertainty proxy is incorporated in a more complex model of a decision sequence (exploration boni are sensitive to the transition type and are ultimately assigned to S1 state-action pairs). In this way higher sum scores of given counters associated with a particular S1 action are incorporated in a simplified, yet still model-based way. Moran and colleagues (2019) have furthermore used similar formalizations to describe interactive dynamics and partial overlap of the proposed MB and MF system. The authors provide evidence for the incorporation of rather parsimonious MF-like value estimates (via sum scores) to retrospectively assign credit to previous actions using an internal model of the environment.

##### Uncertainty estimates based on a trial-counter heuristic

For models *QL+ TRIAL* and *QL + TRIAL + HOP* we followed the same logic, with the only difference being that participants were assumed to utilize a trial counter (*t_n,t_*) as a proxy for uncertainty. This counter heuristic was simply defined as the number of trials since that particular second-stage option was last sampled. The resulting exploration bonus was thus defined as follows:


11 \[
\begin{array}{l} e{{b}_{trial}}\left({{s}_{A}},\ {{a}_{j}}\right)\ =& P\left({{s}_{B}}\ \vert\ {{s}_{A}},{{a}_{j}}\right)\ {\sum}_{a}{{t}_{n,t}}\left({{s}_{B}},\ a\right) \\ & +\ P\left({{s}_{C}}\ \vert\ {{s}_{A}},\ {{a}_{j}}\right)\ {\sum}_{a}{{t}_{n,\ t}}\left({{s}_{C}},\ a\right). \end{array}
\]

In order to match the numerical range to that of the bandit-heuristic described above, counter values for this heuristic were log-transformed. We set the lower bound to 0, so that only non-negative values were considered. Analogous to model Q + BANDIT, action probabilities for first-stage choices were modelled according to Equation 16. The adaptations to yield model Q + BANDIT + HOP were applied here as well, resulting in variant Q + TRIAL + HOP (Equation 17).

##### Habitual Controller

In addition to the trial-wise updating of Q-values and exploration heuristics, the inclusion of Higher Order Perseveration (HOP) also encompasses continuous tracking and updating. In contrast to the subjective value and uncertainty estimates, here, updates relate to subjects’ own history of S1 choices (i.e. are decoupled from reward values and previous S2 actions).

For the QL + HOP model, instead of *rep*(*a*), we included a *habitual controller* (*H_t_*), which accounts for perseveration behaviour in a temporally extended way (Miller et al., 2019). Not dissimilar to previously described TD-learning processes (c.f. Eq. 1 & 2), the habit strength of each choice option is updated according to:


12 \[
{{\text{H}}_{t}}\ =\ {{\text{H}}_{t-1}}\ +\ {{\alpha}_{HOP}}\ \text{*} \ \left(\text{rep}{{\left(\text{a}\right)}_{i,t}}\ -\ {{H}_{t-1}}\right),\]

where rep(a)_*i,t*_ is the same indicator function used in the basic FOP model variants (c.f. Eq. 13 below). If a S1 choice is repeated on the subsequent *rep*(*a*) equals 1 and 0 otherwise. The parameter *α_HOP_* serves as the updating parameter, which determines the extent to which the current choice “overwrites” the previous choice history (i.e. resulting habit strength). This way, the HOP formulation used here includes the FOP variant as a special case for *α_HOP_* = 1. In contrast, values closer to 0 would result in slower updating of habit strength, and thus, indicate a stronger (i.e. longer lasting) influence of past choices.

#### Choice Rules

The standard SoftMax function (*SM*, McFadden, 1973; Sutton & Barto, 2018) served as the basis for all choice rules. According to this rule, choice probabilities scale with the value differences between options.

##### SoftMax

As proposed by Otto and colleagues (2013b), separate coefficients for *Q_MF_* and *Q_MB_* were used, rather than a single weighting parameter *ω* (as done e.g. in Daw et al., 2011 and Gillan et al., 2016). Thus, in the QL + FOP model choice probabilities for action *a* at the first stage were modelled as:


13 \[
\begin{array}{*{35}{l}} P\left({{a}_{i,t}}\ =\ a\ |\ {{s}_{1,t}}\right)\ = \\ \frac{exp\left[{{\beta}_{MB}}{{Q}_{MB}}\left({{s}_{1,t}},\ a\right)\ +\ {{\beta}_{MF}}{{Q}_{MF}}\left({{s}_{1,t}},\ a\right)\ +\ {{\beta}_{persev}}rep(a)\right]}{{\sum}_{a^{\prime}}exp\left[{{\beta}_{MB}}{{Q}_{MB}}\left({{s}_{1,t}},\ a^{\prime}\right)\ +\ {{\beta}_{MF}}{{Q}_{MF}}\left({{s}_{1,t}},\ a^{\prime}\right)\ +\ {{\beta}_{persev}}rep\left(a^{\prime}\right)\right]}. \\\end{array}\]

The parameter ß_*persev*_ describes the “stickiness” of first stage choices, i.e. FOP. As described above, the indicator function *rep*(*a*) equals 1 if the first-stage choice of the previous trial is repeated and 0 otherwise.

Action probabilities follow the same SoftMax as in Equation 13 above, with the only difference of replacing the indicator function from FOP models with the habit vector H_*t*_, so that:


14 \[
\begin{array}{*{35}{l}} P\left({{a}_{i,t}}\ =\ a\ |\ {{s}_{1,t}}\right)\ = \\ \frac{exp\left[{{\beta}_{MB}}{{Q}_{MB}}\left({{s}_{1,t}},\ a\right)\ +\ {{\beta}_{MF}}{{Q}_{MF}}\left({{s}_{1,t}},\ a\right)\ +\ {{\beta}_{persev}}{{H}_{t}}(a)\right]}{{\sum}_{a^{\prime}}exp\left[{{\beta}_{MB}}{{Q}_{MB}}\left({{s}_{1,t}},a^{\prime}\right)\ +\ {{\beta}_{MF}}{{Q}_{MF}}\left({{s}_{1,t}},\ a^{\prime}\right)\ +\ {{\beta}_{persev}}{{H}_{t}}\left(a^{\prime}\right)\right]}. \\\end{array}\]

At the second stage, choices are driven by MF Q-values only (*Q*_2_ (*s_2,t_, a*), as described above) scaled by the second-stage inverse temperature parameter ß_2_, such that:


15 \[
P\left({{a}_{i,t}}\ =\ a\ |\ {{s}_{2,t}}\right)\ =\ \frac{exp\left[{{\beta}_{2}}{{Q}_{2}}\left({{s}_{2,t}},\ a\right)\right]}{{\sum}_{a^{\prime}}exp\left[{{\beta}_{2}}{{Q}_{2}}\left({{s}_{2,t}},\ a^{\prime}\right)\right]}\]

The two baseline models QL + FOP and QL + HOP used these basic versions of the SoftMax. These were extended by incorporating terms that account for directed exploration in first stage choices.

##### Exploration Bonus (eb)

For all FOP variants accounting for directed exploration, *eb* was included in the standard SoftMax and weighted by an additional free parameter *φ*, resulting in the following first-stage choice probabilities (for models including either *eb_bandit_* or*eb_trial_*):


16 \[
\begin{array}{*{35}{l}} P\left({{a}_{i,t}}\ =\ a\ |\ {{s}_{1,t}}\right)\ = \\ \frac{exp\left[{{\beta}_{MB}}{{Q}_{MB}}\left({{s}_{1,t}},a\right)\ +\ {{\beta}_{MF}}{{Q}_{MF}}\left({{s}_{1,t}},a\right)\ +\ {{\beta}_{persev}}rep(a)\ +\ \phi eb\left({{s}_{1,t}},a\right)\right]}{{\sum}_{a^{\prime}}exp\left[{{\beta}_{MB}}{{Q}_{MB}}\left({{s}_{1,t}},a^{\prime}\right)\ +\ {{\beta}_{MF}}{{Q}_{MF}}\left({{s}_{1,t}},\ a^{\prime}\right)\ +\ {{\beta}_{persev}}rep\left(a^{\prime}\right)\ +\ \phi eb\left({{s}_{1,t}},\ a^{\prime}\right)\right]}. \\\end{array}\]

Note that the analogue HOP models (i.e. Q + BANDIT + HOP & Q + TRIAL + HOP) follow a parallel formalisation of S1 choice probabilities when replacing the indicator function for FOP (*rep*(*a*)) with the habit vector, resulting in:


17 \[
\begin{array}{*{35}{l}} M{{F}_{diff}}\ =\ \left[{{P}_{common}}_{,\ rewarded}\ +\ {{P}_{rare}}_{,\ rewarded}\right]-\left[{{P}_{common}}_{,\ unrewarded}\ +\ {{P}_{rare}}_{,\ unrewarded}\right] \\\qquad\qquad\quad \text{and}\ M{{B}_{diff}}\ =\ \left[{{P}_{common}}_{,\ rewarded}\ +\ {{P}_{rare}}_{,\ unrewarded}\right]-\left[{{P}_{common}}_{,\ unrewarded}\ +\ {{P}_{rare}}_{,\ rewarded}\right].\end{array}
\]

#### Hierarchical Bayesian Modelling Scheme

Table 1 provides an overview of all free and fixed parameters for the models described above. Using a hierarchical Bayesian modelling scheme, subject parameters were drawn from shared group-level Gaussian distributions. This resulted in two additional free parameters *M^x^* and *Λ^x^* for each subject-level parameter *x*. Group-level parameter means (*M^x^*) were assumed to be normally distributed (*M* = 0, *SD* = 10) and standard deviations *Λ^x^* were set to follow a uniform distribution (with limits 0 and 10 for all *α^i^*, and an upper limit of 20 for remaining group-level SD parameters). All learning and decay rates (*α*_1_, *α*_2_, *α*_3_, *α_HOP_*) were then back-transformed to the interval (0, 1) using STANs built in cumulative density function. This was done directly within the model, so that raw subject-level parameter values ranging from –10 to 10 were mapped onto the interval (0, 1).

**Table 1:** Free and fixed parameters for all models.


MODEL	FREE PARAMETERS

Q	*α, α*_2_, *α*_3_, ß_*MB*_, ß_*MF*_, ß_*persev*_, ß_2_

Q + BANDIT	*α, α*_2_, *α*_3_, ß_*MB*_, ß_*MF*_, ß_*persev*_, ß_2_, *φ*

Q + TRIAL	*α, α*_2_, *α*_3_, ß_*MB*_, ß_*MF*_, ß_*persev*_, ß_2_, *φ*

Q + HOP	*α, α*_2_, *α*_3_, *α_HOP_*, ß_*MB*_, ß_*MF*_, ß_*persev*_, ß_2_

Q + BANDIT + HOP	*α, α*_2_, *α*_3_, *α_HOP_*, ß_*MB*_, ß_*MF*_, ß_*persev*_, ß_2_, *φ*

Q + TRIAL + HOP	*α, α*_2_, *α*_3_, *α_HOP_*, ß_*MB*_, ß_*MF*_, ß_*persev*_, ß_2_, *φ*


*Note*. Q refers to the basic hybrid model with a FOP term. BANDIT/TRIAL = added first-stage exploration bonus based on respective counter heuristic (c.f. Computational Models section); *φ*: parameter that scales the exploration bonus. Note that this parameter remains the same for both exploration bonus variants, regardless of the specific formalisation of uncertainty estimates in a given model.

All models were implemented using the *STAN* modelling language (version 2.21.0; Stan Development Team, 2020) running in the statistical program R, which was also used for all further analyses (version 3.6.1, R Core Team, 2019). The sampling for each model was done using a Markov-Chain-Monte-Carlo (MCMC) algorithm (no-U-turn sampler NUTS), with four chains running 10000 iterations each, 8000 of which were discarded as warm-up. MCMC methods are based on the generation of a random number sequence (chain) that is used to sample a probability distribution. Parameters estimates with higher (posterior) probability are sampled more often, resulting in a posterior probability distribution. The desired state is reached when a chain has reached equilibrium. \[
\hat{R}\] is a measure of convergence across chains, indicating the ratio of between-chain to within-chain variance. Here, values of \[
\hat{R}\ \le \ \text{1}.\text{1}\] were considered acceptable. Using the default settings of the sampling command, initial values for all parameters were randomly drawn from an interval [–2, 2].

In a first step, all models were compared regarding their predictive accuracy. As this method only provides a relative comparison between models, posterior predictive checks were performed to gain a deeper understanding. These allow a more detailed insight with regard to predictions made by the model and their ability to accurately portrait the data as well as an indication of possible model misspecifications (Wilson & Collins, 2019).

##### Open Code

STAN model code files will be shared via the *Open Science Framework* upon publication. By making the model code freely available, we wish to facilitate further application and development of this model and further adaptations thereof. Transparently reporting on model specifications also holds the potential of direct comparisons of parameter estimates (e.g. as reported above for the data sets compared here). Additional information on the alternative model variants not presented here will be made public upon reasonable request.

##### Open Data

Both data sets analysed here are freely available (for links see the respective publications).

#### Model Comparison

Model comparison was performed using the *loo* package (Vehtari, Gabry, Magnusson, Yao, Bürkner, Paananen &, Gelman, 2023) which provides a measure of predictive accuracy via leave-one-out cross-validation (LOO; Vehtari, Gabry, & Gelman, 2017). To this end the estimated log pointwise predictive density (–elpd) is applied as the criterion of interest. The model with the lowest –elpd score was selected as the best fitting model. While lower values indicate a superior fit, in direct comparisons between models values close to 0 indicate superior fit. As can be seen in Table 3, in these cases the difference in elpd compared to the winning model (*elpddiff*) is provided (thus, values close to zero show “smallest” distance to the winning model). In cases in which a more parsimonious model showed overlap with the best-fitting model in terms of the SE of the elpddiff, the more parsimonious model was chosen. As an additional indicator for model goodness-of-fit point estimates of the *widely applied information criterion* (WAIC) are also reported.

##### Narrowing the Model Space

Our initial model space also included an alternative Bayesian Learning account which was however, omitted due to relative inferior fit across both data sets. In this first phase of model comparisons, we compared Q-Learner and Bayesian Learner models. Due to the high number of possible combinations if including all proposed extensions, the first round of comparisons only included FOP model variants. As the results clearly favoured the standard Q-Learner (Supplement Figure S1), further HOP extensions (and combinations with exploration components) were performed with this better-suited learning formalisation.

## Results

### Model-Agnostic Analyses

In a first step, we quantified MF and MB contributions using common model-agnostic procedures (see e.g. Daw et al., 2011; Otto et al., 2013a; Gillan et al., 2016) as outlined in the methods section. A linear mixed model of S1 stay-switch behaviour using the factors reward and transition type as well as their interaction confirmed the standard effects (Daw et al., 2011; Otto et al., 2013a): in both data sets (see Table 2) we observed a main effect of reward (reflecting MF control) and a reward × transition interaction (reflecting MB control).

**Table 2:** Results from regression analyses of S1 choice repetition probability.


		ESTIMATE	95% CI	z-VALUE	p-VALUE

data1	Intercept	1.35	[1.12; 1.58]	11.48	<.01

	Reward	0.11	[0.05; 0.18]	3.47	<.01

	Transition	–0.07	[–0.14; –0.01]	–2.14	.03

	Reward*Transition	0.47	[0.36; 0.59]	8.10	<.01

data2	Intercept	1.73	[1.53; 1.94]	16.68	<.01

	Reward	0.64	[0.51; 0.77]	9.89	<.01

	Transition	0.02	[–0.03; 0.08]	0.81	.42

	Reward*Transition	0.16	[0.07; 0.24]	3.76	<.01


*Note*. Reward: main effect of reward type (unrewarded vs. rewarded), commonly interpreted as an indicator for MF control; Transition: main effect of transition type (rare vs. common); Reward*Transition: interaction of Reward and Transition type, commonly interpreted as an indicator for MB control.

**Figure 2:**
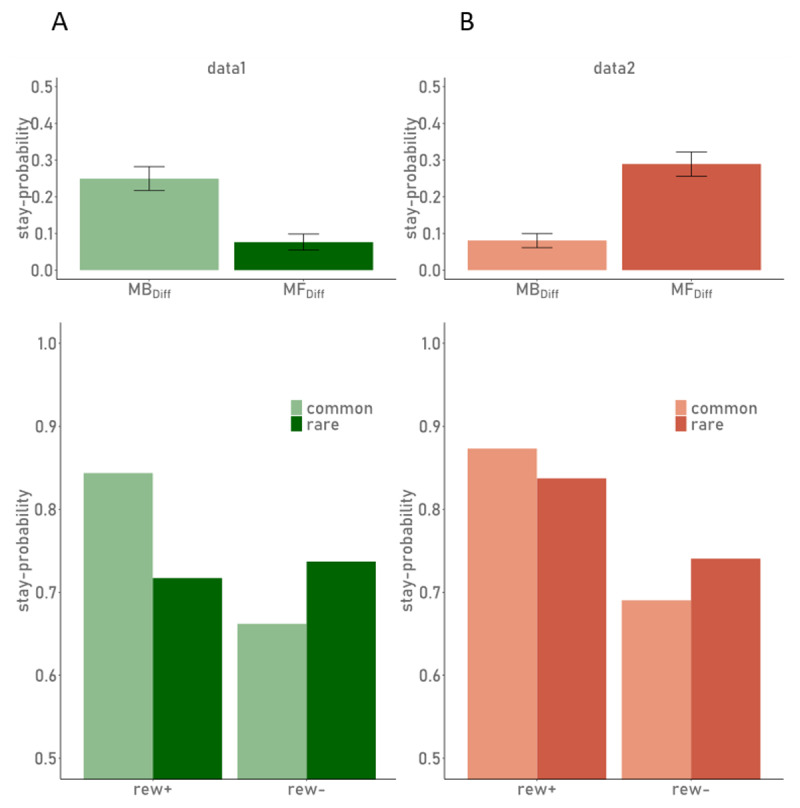
Stay-Probabilities of S1 choices and difference scores. Upper panel: MB and MF difference scores as defined by Eppinger et al. (2013), bar heights depict mean scores over all participants, error bars show the standard error. Lower panel: Probabilities for S1 choice repetition as a function of reward (rew+: rewarded; rew-: unrewarded) and transition type (common/rare) of the preceding trial. The left plots (green, A) show results from data1; the right plots (orange) show results from data2.

However, regression results along with visual inspection (Table 2, Figure 2) also suggest potential differences between task versions (i.e. between data1 and data2). The MF effect was somewhat more pronounced in the data set from Gillan and colleagues (2016; data2), while data1 showed a more pronounced MB effect. This contrast between data1 and data2 was also clearly evident in the respective model-agnostic difference scores for the two effects (Figure 2, lower panel). These differences are however, purely descriptive, due to the different study settings and experimental details, which preclude us from directly comparing effects between studies.

#### Model Comparison

**Figure 3:**
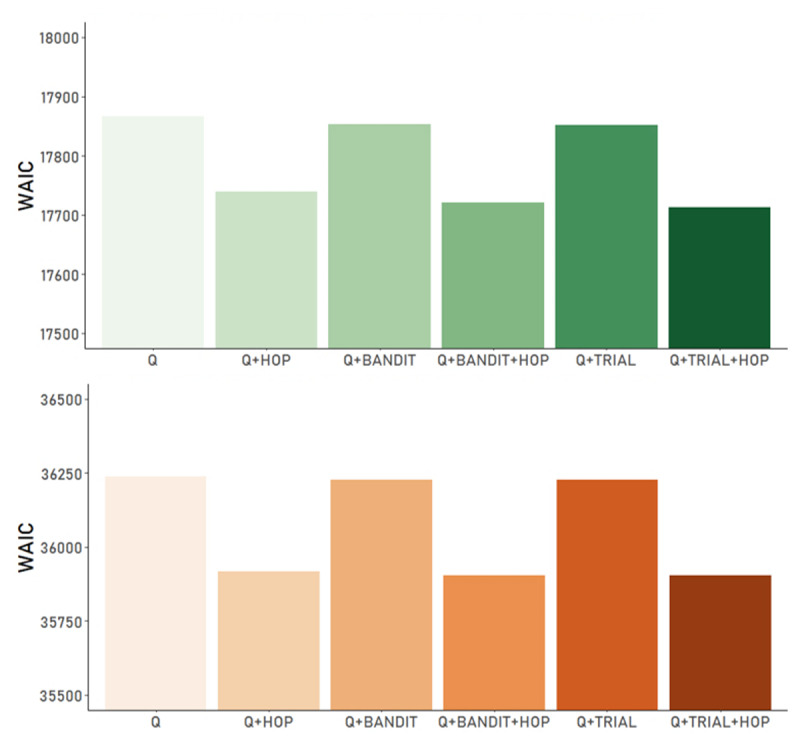
*Model Comparison Results via the Widely Applied Information Criterion (WAIC) for all Q Models (c.f. Table 1*). The upper/lower panel (green/orange bar plots) refer to data1 and data2, respectively. *Bandit/Trial* refer to the model variants with added heuristic-based exploration bonus using stimulus identity/recency, respectively. HOP: model variants with higher order perseveration term; all other versions use a classic FOP term instead (Q, Q+BANDIT, Q+TRIAL).

In both data sets all parameters (group- as well as subject-level) could be estimated well, as evidenced by the aforementioned convergence measure \[
\hat{R}\] (all \[
\hat{R}\ \le \ \text{1}.\text{1}\]). Model comparison then was based on the estimated log pointwise predictive density (–elpd). Here, lower absolute values reflect a superior fit. The difference in –elpd *(–elpddiff*; c.f. Table 3) is provided in reference to the best-fitting model (which itself thus, always has an *–elpddiff* of zero). As outlined previously, the Q-Learner models consistently outperformed BL models across both data sets (cf. Figure S1). Models that include a HOP term outperformed all parallel variants accounting for FOP only (Figure 3).

**Table 3:** Results from model comparison of QL-models with a HOP extension using leave-one-out cross-validation (LOO).


DATA SET	MODEL	*–elpd_diff_*	*se_diff_*	*WAIC*

data1	Q + HOP	–28.9	9.6	17750.21

	Q + BANDIT + HOP	–4.0	6.2	17715.03

	Q + TRIAL + HOP	0.0	0.0	17714.46

data2	Q + HOP	–13.8	8.3	35905.27

	Q + BANDIT + HOP	–11.2	6.2	35887.17

	Q + TRIAL + HOP	0.0	0.0	35871.03


*Note*. The difference in the expected log pointwise predictive density (*elpddiff*) and standard error of the difference (*sediff*). These values show the results of a model comparison using LOO estimates. Each model is compared to the preferred model Q + TRIAL + HOP), as there is no difference between the best-fitting model and itself, values in the first column are always zero.

Within the group of HOP models the variants including an exploration bonus based on a counter heuristic (Q+ BANDIT/TRIAL + HOP) was numerically superior in both data sets (Table 3). However, compared to the Q+HOP model, these differences in goodness of fit (elpd & WAIC) were too small to provide definitive, robust evidence for an added benefit when considering both data sets.

According to some scholars a more complex nested model is warranted if the posterior estimate of an additional parameter in question (i.e. here *φ*) is reliably different from 0 (see e.g. Kruschke, 2011). While this is the case for both data sets (c.f. 95% HDI of *φ*; Table S1), for the main text we are focusing on the reduced model variant (QL+ HOP), based on the inspection of the ratio of benefit in predictive density and its uncertainty (i.e. *elpddiff/sediff*; c.f. Table 3).

Following common conventions (Vethari et al., 2017; Vethari et al., 2023) we thus, interpret the evidence in favour of model Q + TRIAL + HOP (vs. the simpler Q + HOP) in data1 as moderate (28.9/9.6 = 3.01) in data2 however, only as weak (13.8/8.3 = 1.66). Additionally, the more straight-forward interpretation of model parameters as well as our overarching aim of providing a comprehensive and easily applicable refinement of standard hybrid models resulted in the decision for the more parsimonious model version.

Note however, that model fits for data1 were notably improved by inclusion of the exploration term, which was the case regardless of the models’ learning component (i.e. also present in BL and FOP variants, see Figure S2). The marked differences in sample, experimental procedure and task version between data sets may likely explain the specificity of superior model fits due to the inclusion of the exploration mechanism. For completion, therefore, all results from the Q+TRIAL+HOP model for Data1 are provided in the supplement, while the main text will focus on the more parsimonious Q+HOP model.

##### Posterior Predictive Checks

While results from model comparisons can provide relative support for one computational account over another, posterior predictive checks are required to ensure that a model can reproduce core patterns in the data. We thus, simulated 10000 full trial sequences, 8000 of which were discarded as warm-up, yielding 8000 (2000 per chain × 4 chains) simulated data sets per subject. Simulations were performed during fitting the model to the empirical data (i.e. on the basis of possible parameter values sampled during this procedure). As can be taken from Table 4, simulated S1 choices largely reproduced the patterns observed in human data. We repeated the model-agnostic analyses of stay-/switch-behaviour (shown above for empirical data, c.f. Table 2, Figures 2 and 5) for the simulated data sets. Visual inspection (Figure 5) revealed an underestimation of “*stay*”-probabilities (P(stay)) in the simulations when compared to the empirical data. Nonetheless, the overall pattern of stay-switch tendencies as a function of reward and transition were largely reproduced, whereas this was less pronounced for the main effect of reward (MF contribution; Table 2, Figure 2).

**Table 4:** Proportion of correct S1 choice predictions by the winning model Q +HOP.


DATA SET	MIN	25^th^ PERCENTILE	MEDIAN	MEAN	75^th^ PERCENTILE	MAX

data1	.519	.638	.764	.748	.841	.916

data2	.505	.687	.767	.754	.829	.977


*Note*. Summary statistics are based on the comparison of individuals’ choices with model predictions, which were pooled and averaged for each data set.

#### Posterior Distributions

**Figure 4:**
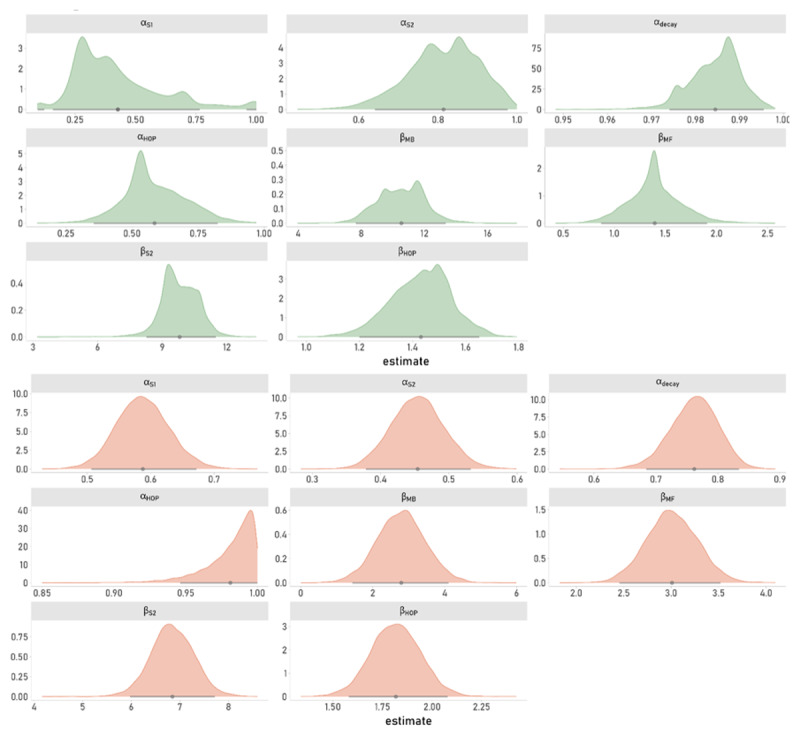
Posterior Distributions of Group-Level Mean Parameters From Model Q + HOP. Solid gray lines show the 95% highest density interval (HDI) and dots depict the point-estimate of the mean. Panels A and B (green and orange plots) show results on the basis of data sets data1 and data2, respectively.

Figure 4 shows the posterior distributions of group-level parameters underlying S1 choices in the best-fitting model. The resulting posterior estimates mirrored the model-agnostic analyses portraited above, showing a descriptively more pronounced MB component (ß_*mb*_) in data1 vs. data2 (Figure 4). The step size parameter *α_HOP_*, also exhibits differences between the two data sets. Again, it should be noted however, that such differences are not directly interpretable due to the distinct and independent experimental settings. It is, nonetheless, interesting to note that the step-size parameter in data2 is estimated to be close to 1, which leads the HOP term to approximate FOP (in the case of *α_HOP_* = 1 only the last step is considered with regard to perseveration). Habit-updating in data1 in contrast, seems to occur in a slower, more gradual fashion, thus, more closely resembling HOP.

**Figure 5:**
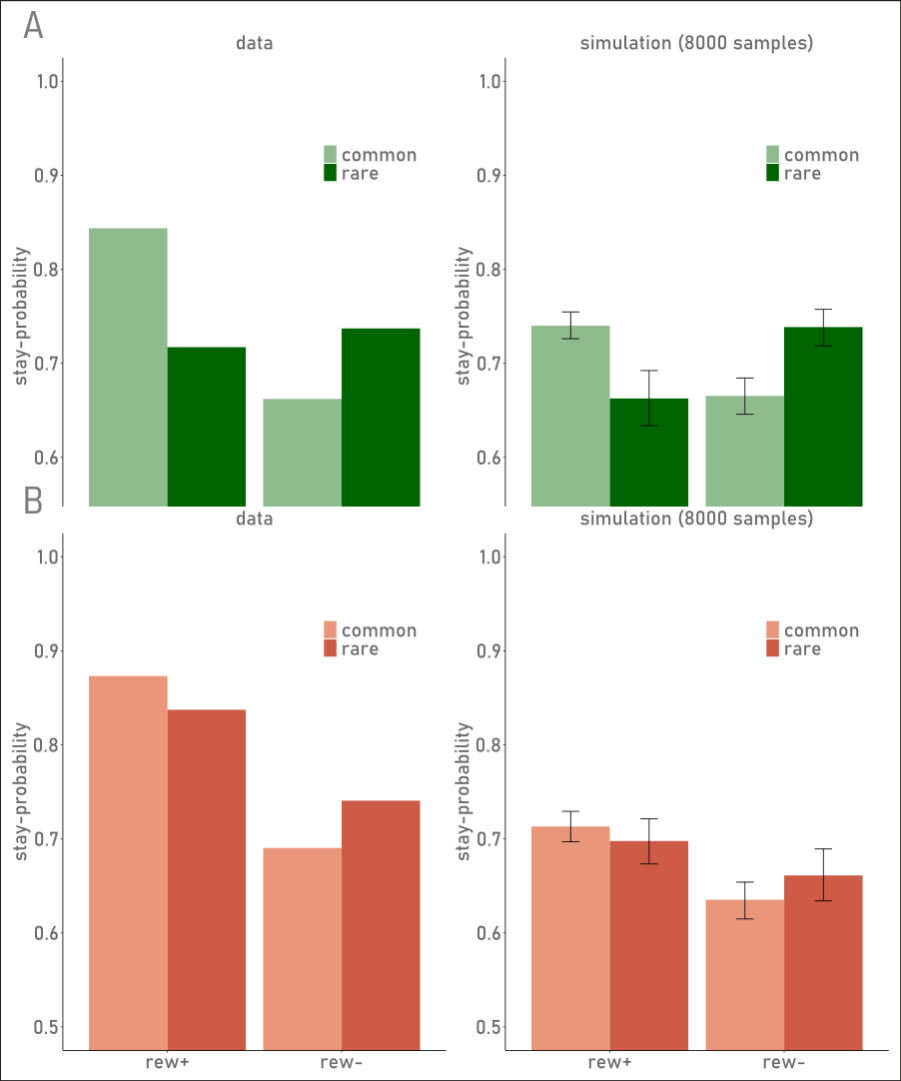
Probabilities of S1 choice repetition as a function of reward and transition type. Y-axis: Stay probabilities for 1st stage choices; data: empirical stay probabilities from data sets data1 (panel A; green) and data2 (panel B; orange). simulation: stay-probabilities from N = 8000 simulated choice sequences per subject, derived from the winning model (Q + HOP).; rew+/–: previous trial was rewarded (+) or unrewarded (–).; common/rare: previous trial followed a common/rare transition, respectively. Error bars in the simulation plots depict the 95% HDI over 8000 simulated data sets.

Posterior point-estimates of all hyperparameter means are shown in Table 5, results from the model extension with the directed exploration term based on the *TRIAL* heuristic can be found in the supplement (Table S1, Figure S2). As mentioned above, results from this extended model indicate that meaningful traces of strategic exploratory behaviour were present in both data sets (group-level mean of the exploration bonus parameter (φ) was positive in both data sets and the 95% HDI did not overlap with 0, Table S1, Figure S2).

**Table 5:** Posterior Estimates of Group-Level Parameters from Model Q + HOP.


PARAMETER	data1		data2	
	
	MEDIAN_x_	95%HDI	MEDIAN_x_	95%HDI

*α*_1_	0.38	[0.10, 0.83]	0.59	[0.51, 0.67]

*α*_2_	0.82	[0.64, 0.96]	0.45	[0.38, 0.53]

*α*_3_	0.99	[0.97, 1.00]	0.76	[0.68, 0.83]

*α_HOP_*	0.57	[0.35, 0.82]	0.98	[0.95, 1.00]

ß_*mb*_	10.59	[7.65, 13.33]	2.80	[1.44, 4.11]

ß_*mf*_	1.39	[0.87, 1.91]	3.00	[2.46, 3.52]

ß_*HOP*_	1.44	[1.20, 1.65]	1.82	[1.58, 2.10]

ß_2_	9.76	[8.26, 11.49]	6.84	[5.97, 7.72]


*Note*. Posterior point-estimates of hyperparameter medians and corresponding 95% highest density intervals (95%HDI) for data1 and data2 from the winning model (Q + HOP) for all subject-level parameters × listed in the first column.

##### Correspondence with Model-Agnostic Analyses

**Figure 6:**
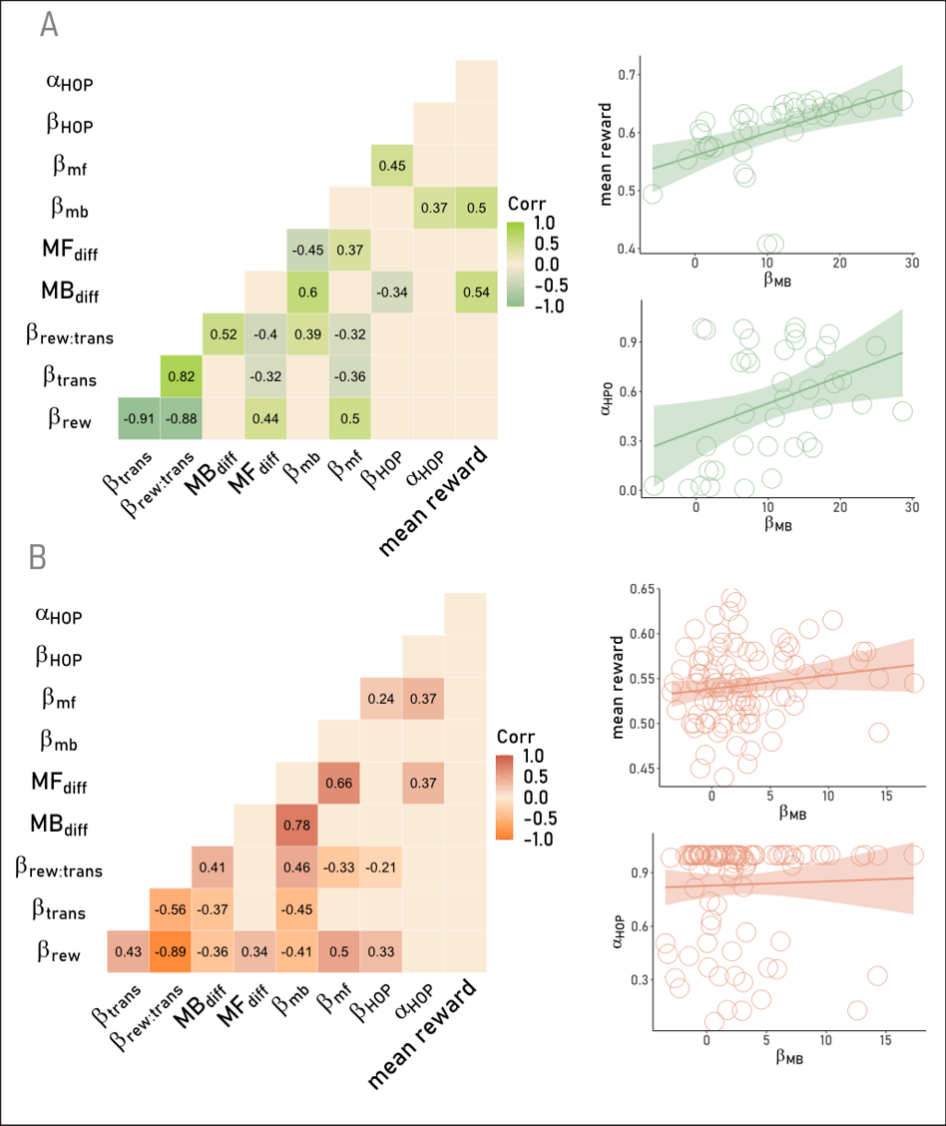
Associations of model-agnostic and model-derived indices of MB and MF control for data1 **(a)** and data2 **(b)**. Empty tiles (left panels) indicate non-significant associations. ß_*rew*_, ß_*trans*_, ß_*rew:trans*_: regression weights for main effects of reward, transition type and their interaction; *MB_diff_*, *MF_diff_*: differences scores of MB and MF influences on S1 stay probabilities respectively; ß_*MB*_, ß_*MF*_: MB and MF S1 choice parameters from the winning model; ß_*HOP*_: S1 higher order perseveration parameter; mean reward: mean reward gained throughout TST (data1: 300 trials, data2: 200 trials). Right panel: association of model-derived MB (ß_*MB*_) and habit step-size parameter ∝_*HOP*_ with mean reward. Circles depict individual participants. Plots in panel A (top row, green) are based on data1, plots in panel B (bottom row, orange) are based on data2.

In order to investigate how parameters derived from the model relate to model-agnostic indices of MB and MF behaviour as well as to the overall performance, correlation analyses were performed (Figure 6). For this purpose, we applied the same regression model as described for the group analyses to each participant’s individual data set, omitting the random effects term, resulting in:


\[
\text{pstay} \sim \text{rew} * \text{trans} + \left(\text{rew} * \text{trans} + 1\right)
\]

Model-agnostic indices of MB (ß_*rew:trans*_ and *MB_diff_*) and MF (ß_*rew*_ and *MF_diff_*) exhibited moderate associations in both data sets (Figure 6). Both effects were likewise associated with the corresponding model-derived parameters (ß_*MB*_, ß_*MF*_). The MB components (ß_*MB*_, *MB_diff_*) showed a moderate to strong association to the mean overall pay-out in data1 but not data2, confirming that the modified version successfully addressed previous concerns (see Kool et al., 2016). In data2, there was no evidence for this association (see Figure 6B). The choice weight parameter for the HOP extension (ß_*HOP*_) was positively associated with the model-derived parameter for MF control across both data sets. In contrast, the second newly introduced parameter, HOP step-size *α_HOP_*, showed a diverging pattern. While positively associated with the model-based MB component in data1, in data2 there was no such relation but instead a positive association with the model parameter for MF control. This qualitative pattern may point to a differentiation in parameter relations when considering FOP (c.f. data2 *α_HOP_* close to 1) vs. HOP.

### Further Validation of the Best-Fitting Model

**Figure 7:**
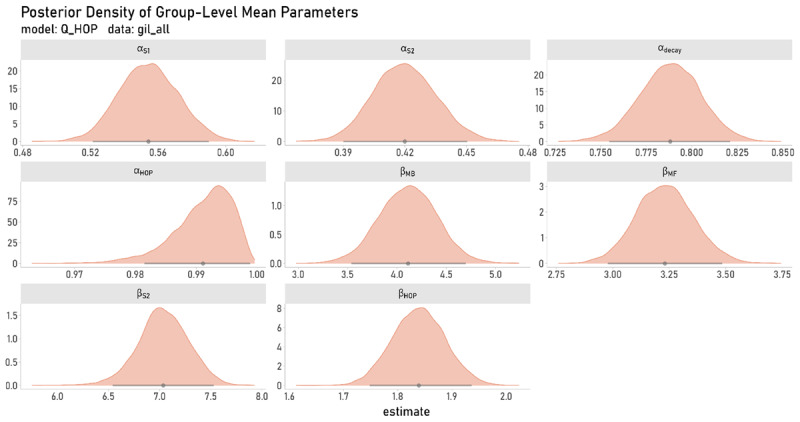
Posterior Density Estimates Based on The Full Sample of Data2. Group-level parameter estimates from model variant Q+HOP derived from fitting the full sample of the original publication (Gillan et al., 2016; N = 548; Experiment 1). The lower panel of Figure 4 shows corresponding results based on data2 (N = 100). Grey dots indicate the mean point-estimate, bars depict the 95%-HDI.

In a final step, we verified that results for data2 were not due to specific characteristics inherent to the subsample we drew from the data set from Gillan et al. (2016). To this end, we repeated the model estimation procedure for models Q + HOP and Q + TRIAL + HOP (c.f. Methods described above) using the full sample of experiment 1 (N = 548) from the original publication. Modelling results were comparable to those reported above for the initial subsample (data2). Both models converged equally well (all \[
\hat{R}\ \le \ \text{1}.\text{1}\]). However, in contrast to the ambiguously small numerical advantage for the exploration model over its reduced version in data2 (c.f. Table 3), this benefit was more clearly pronounced in the full sample, where the Q + TRIAL + HOP model outperformed the Q + HOP variant (Supplement Table S3). The posterior estimates of group-level parameters from this model based on the full sample (N = 548) mirrored results reported above (c.f. Figures 4 and 7).

## Discussion

Here we extended existing hybrid models of TST behaviour with mechanisms implementing directed (uncertainty-based) exploration and value-free perseveration (higher-order perseveration, HOP). To this end, we considered several ways in which uncertainty may guide participants choices in stage 1 of the task, building upon insights from the exploration-exploitation literature (e.g. 4-armed restless bandit; Daw et al., 2006; Chakroun et al., 2020; Wiehler et al., 2021), as well as first-order vs, higher-order choice repetition effects (References see above). While the TST does not clearly decouple reward and information (in contrast to more specific tasks designed to study exploration, e.g. Wilson et al. 2014), the aim of the present study was to examine potential benefits of incorporating exploration and perseveration mechanisms in models for one of the most widely-used tasks in computational psychiatry.

An uncertainty-dependent learning model (Bayesian learning models using a Kalman Filter) did not provide an advantage over a classic Q-Learner algorithm. We generally observed a positive heuristic-based exploration effect for stage 1 of the task, but the improvement in model fit was only reliable in some analyses (e.g. data1 and full but not reduced sample of data2), and the effect was generally small, such that our primary analyses focused on models without directed exploration terms. In contrast, the inclusion of HOP yielded clear benefits across both independent data sets, which applied two different versions of the TST assessed in different settings (laboratory vs. online sample).

We complemented model-based results with more traditional model-agnostic analyses to gain a deeper understanding of how these relate to each other. This additionally aided the interpretation of the best-fitting model, parameter estimates derived from it as well as qualitative differences in the results obtained from both data sets.

### Uncertainty-based exploration on the TST

While human choice behaviour in the restless bandit task is typically better described by models that incorporate a Kalman-Filter as a learning component (vs. a constant learning rate; see e.g. Daw et al., 2006; Raja Beharelle et al., 2015; Chakroun et al., 2020; Wiehler et al., 2021), this was not the case for TST data analysed here. This may likely be due to the more complex task structure and resulting higher cognitive demands. Thus, tracking the underlying reward walks and uncertainties associated with them might be too computationally demanding.

Participants exhibited evidence for uncertainty-dependent exploration effects on S1 choice behaviour. Specifically, exploration bonus parameters were generally reliably >0 across data sets and model variants, dovetailing with previous results using restless bandit tasks (Chakroun et al., 2020; Speekenbrink 2022; Wiehler et al., 2021). At the same time, model comparison in some cases failed to provide conclusive evidence in favour of the inclusion of directed exploration terms (see e.g. the N = 100 subsample for data2), leading to a somewhat inconclusive situation where a nested model parameter was clearly different from zero (indicating a positive effect of directed exploration on S1 choice behaviour), and model comparison was partly inconclusive. Nonetheless, this suggests that the strategic utilisation of uncertainty in TST choice behaviour should be considered in future investigations, while our results also suggest that these effects in TST S1 choice behaviour may be overall consistent, but likely small.

### Perseveration Behaviour on the TST

Next to the employment of uncertainty, participants’ propensity for perseveration and MB control seem to also afford more nuanced investigation in the context of TST data (and beyond).

The present results show a clear improvement in model fit across both task versions and study set-ups due to the inclusion of S1 HOP behaviour. Descriptively, however, results for data1 and data2 differed. In light of the pivotal role of habits in common theories of addiction (c.f. Everitt & Robbins, 2005; Voon et al., 2017), a more nuanced definition and formalisation is called for (see e.g. Nebe et al., 2024). Repetitive behaviours that are based on e.g. one’s own choice history (i.e. HOP) may serve to stabilise behaviour over time. On the other hand, perseveration may also stem from skewed or erroneous value-estimates or lack of behavioural flexibility. Accounting for HOP therefore leads to cleaner process estimates for other mechanisms included in a model.

Though only descriptive in nature at this point, based on the qualitative, diverging pattern in present results, more FOP-like behaviour, as seen in data2 (i.e. step-size close to 1; classic TST version) seems to be associated with MF mechanisms. The analogous, however, more HOP-like behavioural pattern in data1 (i.e. step-size close to 0.5; modified TST) in contrast, showed association with indices of MB control. These findings suggest that the dynamics and associations of decision-making subcomponents may be of interest for our understanding of potential dysregulations along the mental health spectrum. A computational modelling approach as done here, can potentially help to tease apart facets of perseveration.

The potentially differential employment of MB control in data1 and data2 may also be viewed in light of factors specific to each study and the resulting utility of this strategy. As the instructed goal for participants in both studies was to maximize their pay-outs, placing higher priority on the MB system might be seen as clearly advantageous. As Kool and colleagues (2016) have pointed out, such an advantage is however, dependent upon the task version at hand. This finding is supported by the significant positive association of MB control and rewards earned in data1 (modified version of the TST) and a lack thereof in data2 (classic TST version). These differential associations also show that previously voiced criticisms and proposed alterations (Kool et al., 2016) have successfully been addressed in the adapted task version used in data1. Thus, relatively lower MB behaviour in data2 may even be seen as goal-directed in a broader sense, as MB control is commonly viewed as more demanding, while in this case not more rewarding and ultimately too costly.

The assessment within a laboratory setting further enabled more control over participants’ understanding of the task at hand compared to the online sample from which we derived data2. As several scholars have pointed out over the past years, general task understanding and diverging instructions of the TST can have a significant influence on the relative employment of the MB over MF system (e.g. Akam et al., 2015; Feher da Silva & Hare, 2018; Castro-Rodrigues et al., 2022; Hamroun, Lebreton,& Palminteri, 2022). It should be noted that the authors of the original publication of data2 have taken extensive precautions such as training trials and a comprehension test (for details see Gillan et al., 2016) prior to execution of the TST. Nonetheless, insight into participants’ model of the task along other aspects such as motivation, situational external influences etc. remains reduced within this online context. Noticeable differences in the compensation and therefore incentive for participation may have further influenced individuals’ motivational state with regard to more effort placed on task execution (see e.g. Patzelt et al., 2019).

### Dual-System Views and the TST

Beside these external influences on relative MB and MF contributions (i.e. task versions, instructions, incentives etc.) broader criticisms regarding their definition within classic dual-system frameworks has been raised. As outlined previously, indices of MB control likely only depict one possible goal-directed strategy subjects use to complete the TST (recall reduced MB control in data2 vs. data1 in light of its utility for reward maximization, i.e. long-term goal-attainment). Consequently, several alternative strategies that may also utilize a model of the environment, rendering them MB in the literal sense, are not accounted for (Feher da Silva & Hare, 2018; 2020; Toyama et al., 2017; 2019). Models employed on the other hand, may be skewed, outright incorrect, or employed in a rigid and habitual way, further complicating a clear-cut interpretation of associated indices (see e.g. Seow et al., 2021; Shahar et al., 2019a). The same holds true for potential additional subprocesses which are not represented in classic dual-system views and formalizations thereof (Collins et al., 2017; Collins & Cockburn, 2020; Feher da Silva et al., 2022). At this point it should be noted that several of these issues may also apply to the explore-exploit research and theoretical assumptions works in this field are based on.

To address these concerns several scholars have been developing adapted versions or novel alternatives to these paradigms (see e.g. Kool et al., 2016 and the adapted TST version applied by Mathar et al., 2022; Bruder et al., 2021; Wagner et al., 2022 etc.). To name one prominent example posed as an alternative (or at the least useful supplement) to widely applied classic restless bandit paradigms in the explore-exploit research, Wilson and colleagues (2014) have introduced the *Horizon Task*. As alluded to previously, this paradigm is aimed at the decoupling of reward and information, which are classically confounded and thus, hamper the clear distinction between exploration, exploitation and their driving factors. The Horizon Task has been successfully applied in a number of studies and has thus far also undergone several further adaptations (e.g. Feng et al., 2021; Cogliati Dezza et al., 2017; Sadeghiyeh, et al., 2020).

### Computational Modelling

Another complementary approach is the development of more precise computational models to better delineate the specific processes engaged during task performance. One recent example for such efforts comes from Gijsen, Grundei, and Blankenburg (2022): The authors applied an active inference account to TST data and – akin to the procedure laid out here- re-analysed existing data sets, some of which were better accounted for by the proposed more elaborate models. However, specifically data gathered in an online setting as well as data including a negative reinforcement scheme exhibited differences in model ranking. The preferred model for these data sets (referred to as *online* and *shock* data sets respectively in Gijsen et al., 2022) was in fact more akin to versions tested here. Moreover, it should be noted that the current study followed a different aim in more general terms. As pointed out previously, the TST is arguably the most widely-used paradigm to capture MB and MF behaviour. By extending existing models that are already in use, we hope to balance improving their descriptive ability while at the same time ensuring their applicability for a wide scientific audience. By making the code for the best-fitting model freely available, we hope to foster similar efforts (c.f. *Open Code* above).

### Limitations of the current study

Common to all computational modelling approaches are basic considerations regarding the limited scope of possible mechanisms accounted for. Results derived from computational models (and their comparison) are ultimately limited to the finite set of processes defined in them. Due to its non-specific applicability, this issue may almost seem trivial, but should nonetheless be kept in mind when evaluating and interpreting such results. For example, alternative implementations of exploration and/or additional processes not accounted for here could be taken into account in future investigations.

In addition to the broader conceptual issues with regard to the TST, more specific limitations of the present study should be noted as well. While results from model comparison clearly favoured all QL over BL models in both data sets, the implemented belief updating process in the latter model family is an approximation (vs. exact representation) of the true underlying random walk dynamics (control analyses however showed that the empirical reward dynamics closely corresponded to those implemented in all BL models). Note that the Kalman-Filter updating process between trials (Supplement Equation 4) in all BL models is analogous to the *forgetting* process implemented in the QL model variants (Equations 4 and 5). Thus, for both learning mechanisms we assumed subjective value estimates of unchosen options to move closer to a reasonable estimate (mid-range of possible values). Nonetheless, future applications may consider refining this model aspect.

As discussed above, despite the fact that directed exploration estimates were consistently positive across data sets and models, these effects were numerically small. In addition, model comparison (e.g. Q + TRIAL +HOP vs. Q + HOP) did not unequivocally favour the exploration variants when considering both data sets and data2 subsamples. Model Q + TRIAL + HOP yielded a reliably superior fit when considering the full sample (N = 548, c.f. Table S3), the lack of a reliable benefit in the subsample (data2; N = 100) suggests that exploration effects in TST data (in particular the classic TST version assessed in an online study) may be numerically small.

To our knowledge, model simulations analogous to the posterior predictive checks carried out here are currently not available from related work. This complicates the interpretation of our simulation results. Posterior predictive checks revealed that the best-fitting model (Q + HOP) accounted for the overall data pattern quite well, it still underpredicted S1 stay-probabilities in both data sets (c.f. Table 4 and Figure 4 above). Future work is therefore required to determine the degree to which this depends on the specific task version employed, or reflects a general shortcoming of current hybrid models.

Another potential limitation is that recently applied DDM choice rules were not considered in the present study (Pedersen, Frank, & Biele, 2017). The investigation of reaction time distributions and their relation to information processing and decision-making can provide valuable insights that may complement present results (Shahar et al., 2019b). Parameters derived from models like these have further been linked to various (sub-)clinical symptoms, and thereby also shed light on potential disease mechanisms (see e.g. Forstmann, Ratcliff, & Wagenmakers, 2016; Mandali et al., 2019; Maia, Huys, & Frank, 2017; Sripada & Weigard, 2021). Because our goal was first and foremost to confirm the advantage of proposed model extensions in different TST versions, we leave the application of DDM choice rules to future work.

The empirical data used to develop and test the proposed novel model variants may pose yet another limiting factor. We included two TST versions as a first step, but several additional task variants could be examined in future work in order to further validate the adapted hybrid model. Considering aforementioned ambiguities with regard to the generalizability and transferability of proposed exploration- and perseveration-strategies these should clearly be tested for in further, heterogeneous TST data sets. In order to (at least in part) account for the myriad of contextual factors influencing learning and decision-making in such paradigms, data from within-subject design studies explicitly examining specific contextual effects would be required to delineate how the proposed HOP and exploration mechanisms are modulated by these factors.

An additional issue concerns the generalizability of the present results. A large part of empirical findings is based on small rather homogenous groups of individuals, namely WEIRD ones (i.e. white, educated, industrialised, rich, & democratic), which also applies to many other data sets in the field. Despite participants’ *WEIRDness*, samples are seldomly diverse with regard to age or gender either. In the present case for example, the sample from data1 was exclusively comprised of 18 to 35-year-old heterosexual males (Mathar et al., 2022). While reducing variability in these sample characteristics has its’ utility, (improving internal validity and thus enabling more clear-cut interpretations) results are consequently limited to this confined group. Gillan and colleagues (2016) on the other hand employed a more diverse large-scale community sample. Despite lesser concerns regarding diversity, here other limitations that arise due to the online setting and associated factors come into play (e.g. data quality due to false profiles, low incentives, task understanding etc.).

Despite ever growing popularity and application of transdiagnostic as well as dimensional conceptualisations of mental health, a substantial body of research is still based on the comparison of groups defined as either *healthy* or *diseased*. Again, procedures like this have a rational basis, entail advantages, and have produced a wealth of valuable insights. Keeping this and aforementioned progress in mind (Insel et al., 2010; Robbins et al., 2012; Maia & Frank, 2011), it is nonetheless warranted to push further. Future studies that leverage large samples and the natural sub-clinical variation in psychiatric symptomatology these entail, are called for.

## Conclusion

Here we compared a series of extensions of commonly applied hybrid models for TST behaviour using concepts from the exploration-exploitation literature and work on perseveration behaviour (Miller et al., 2019; Wilson et al., 2021). Results provide computational evidence for a contribution of higher order perseveration to behaviour in two independent data sets of different variants of the a widely used two-step task (TST). A model with a higher order perseveration term for S1 decisions consistently outperformed standard models accounting only for first-order perseveration. Inclusion of a heuristic-based directed exploration term generally yielded positive exploration bonus parameters, similar to related work using other reinforcement learning tasks. However, these exploration effects were overall small, and model comparison was in some cases inconclusive. Future work may extend these approaches to other task variants, and explore the degree to which directed exploration and/or higher-order perseveration effects in TST behaviour are sensitive to e.g. individual differences in (sub-)clinical psychopathology.

## Additional File

The additional file for this article can be found as follows:

10.5334/cpsy.101.s1 Supplement. Additional Modelling Results.
