# Supplementary material for: Signatures of Perseveration and Heuristic-Based Directed Exploration in Two-Step Sequential Decision Task Behaviour
Source: Comput Psychiatr. 2025 Feb 11;9(1):39–62. doi: 10.5334/cpsy.101 (PMC11827566; doi:10.5334/cpsy.101)
Supplement: Supplement. — Additional Modelling Results. [file cpsy-9-1-101-s1.pdf]

**Supplement**

## *Bayesian Learner (BL)*

The second learning rule considered in the initial model comparisons is based on the *Bayesian Learner* (BL) as commonly applied in restless bandit tasks (Daw et al., 2006; see also: Chakroun et al., 2020; Wiehler et al., 2021). Here, the constant learning rate $\alpha_2$ (for Q-value updates at S2) is replaced by a trial-specific learning rate ($\kappa_t$) based on the Kalman Filter (Kalman, 1960). The idea is that participants track the changes in the underlying reward means of all choice options, as well as the uncertainty associated with these estimates. In this model, learning rates are then uncertainty-dependent.

For both data1 and data2, the true process underlying the Gaussian random walks of S2 rewards was directly incorporated in the model (for applications in the explore-exploit dilemma see e.g. Daw et al., 2006; Chakroun et al., 2020).

For data1, rewards for each option $n$ at trial $t$ ranged between 0 and 1 (multiplied by 100 and rounded to the next integer for presentation as points in the task; c.f. Methods). These were generated following a Gaussian random walk with mean $\mu_{n,t} = 0.5$ , and SD $= 0.04$ (observation variance $\sigma_o^2 = 0.04^2$). Means for each option independently changed on a trial-to-trial basis.

As participants were assumed to represent the reward-walk dynamics in their internal models, the computational model includes fixed parameters $\hat{\gamma}$, $\hat{\theta}, \hat{\sigma}_o, \hat{\sigma}_d$, which are set to values approximating those of the underlying random walk: the decay parameter $\gamma$ = 0.9836, decay centre $\theta$= 0.45, and the diffusion variance $\sigma_d^2$= $0.025^2$ , and diffusion noise $v_t \sim N(0, \sigma_d^2)$ .

Participants start with prior beliefs of a normally distributed reward mean $\hat{\mu}_{n,t}^{pre}$ with variance $\hat{\sigma}_{n,t}^{2pre}$ of a chosen second-stage option $n$ on trial $t$ and update these in light of the reward $r_t$ they receive according to:

(1) $\hat{\sigma}_{n,t}^{2post}$= (1-$\kappa_t$)$\hat{\sigma}_{n,t}^{2pre}$ and

(2) $\hat{\mu}_{n,t}^{post}$=$\hat{\mu}_{n,t}^{pre}$+ $\kappa_t\delta_t$ with $\delta_t$= $r_t$-$\hat{\mu}_{n,t}^{pre}$.

The parameter $\kappa_t$ is the Kalman Gain, which serves as the uncertainty-dependent learning rate, similar to $\alpha_2$ in the original (QL) model. In the updating process of a chosen option the Kalman Gain (just like $\alpha$) scales the prediction error used for updating the mean reward estimate ($\hat{\mu}_{n,t}^{pre}$). The important difference being that $\kappa_t$ varies on a trial-to-trial basis depending on the observation as well as diffusion variance:

(3) $\kappa_t$= $\hat{\sigma}_{n,t}^{2pre}$ / ($\hat{\sigma}_{n,t}^{2pre}$ + $\hat{\sigma}_o^2$).

Thus, high observation uncertainty yields large values of $\kappa_t$ and thus increased updating. In contrast, low observation uncertainty yields small values of $\kappa_t$ and thus reduced updating.

Values of all options are updated between trials according to:

(4) $\hat{\mu}_{n,t+1}^{pre}$= $\hat{\gamma}\hat{\mu}_{n,t}^{post}$ + (1-$\hat{\gamma}$)$\hat{\theta}$ and

(5) $\hat{\sigma}_{n,t+1}^{2pre}$= $\hat{\gamma}^2$ $\hat{\sigma}_{n,t}^{2post}$ + $\hat{\sigma}_d^2$.

That is, between-trial updating describes participants tracking of the assumed dynamics underlying the Gaussian random walks. Due to these between-trial updating dynamics, the forgetting process for S2 Q-values (see Equation 5 main text) was omitted from all BL models.

The Kalman Filter (Equation 12) was implemented as the learning rule for second-stage values and then incorporated into the hybrid model, such that $\hat{\mu}_{n,t}^{pre}$substitutes the Q-values for second-stage options in the SoftMax.

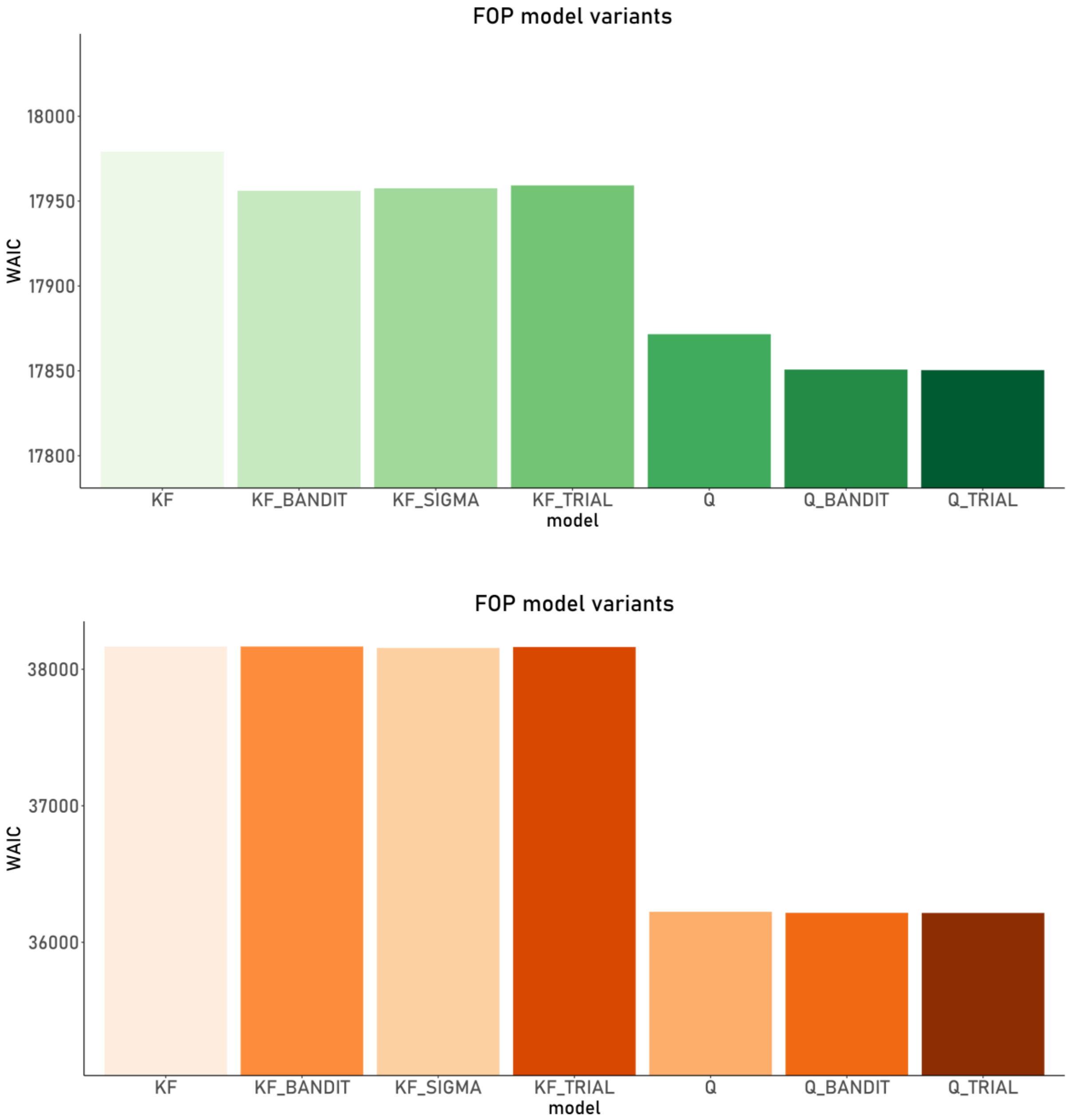


**Figure S1. Comparison of FOP Model Variants.** KF = Bayesian Learner models using a Kalman Filter updating rule. SIGMA/BANDIT/TRIAL = different implementations of an exploration bonus as described in the methods section. All model variants depicted here use the original FOP term. Upper panel/green plots: fit indices are based on data1. Lower panel/orange plots: fit indices based on data2.

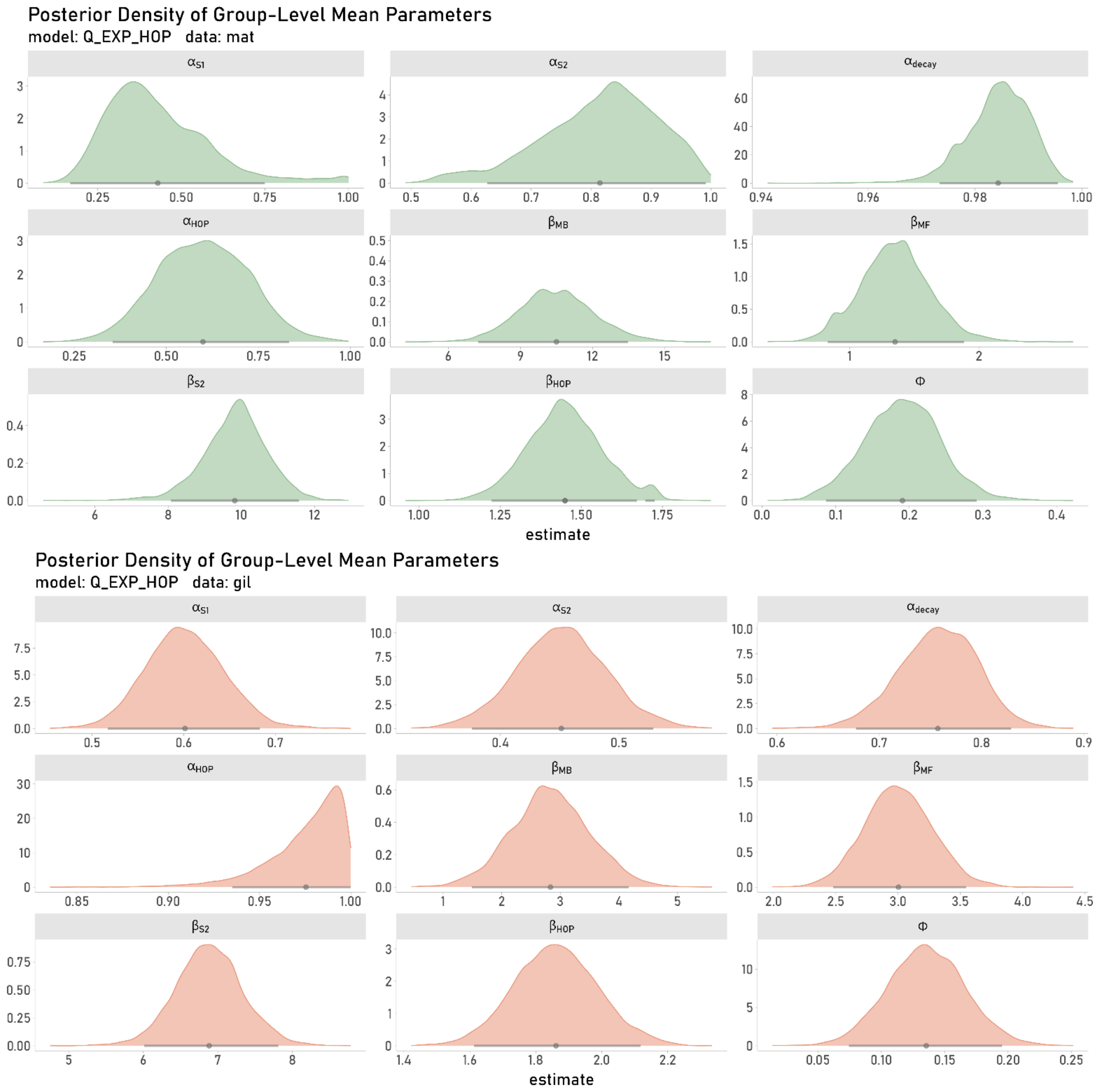


**Figure S2. Posterior Distributions Q + TRIAL + HOP**.Posterior distributions of Group-Level Means of S1 Choice Parameters Based on Model Q + TRIAL + HOP. Solid gray lines show the 95% highest density interval (HDI) and the dot depicts the point-estimate of the mean. Panels A and B (green and orange plots) show results on the basis of data sets data1 and data2, respectively.

**Table S1. Posterior Point-Estimates of Group Level Parameters from the Model Q + TRIAL+ HOP.**

| parameter | data1 | | data2 | |
|---|---|---|---|---|
| | $Median_x$ | 95%HDI | $Median_x$ | 95%HDI |
| $\alpha_1$ | 0.40 | [0.17,0.75] | 0.60 | [0.52,0.68] |
| $\alpha_2$ | 0.83 | [0.63,1.00] | 0.45 | [0.38,0.53] |
| $\alpha_3$ | 0.98 | [0.97,1.00] | 0.76 | [0.68,0.83] |
| $\alpha_{HOP}$ | 0.60 | [0.35,0.83] | 0.98 | [0.94,1.00] |
| $ß_{mb}$ | 10.45 | [7.24,13.47] | 2.82 | [1.50,4.18] |
| $ß_{mf}$ | 1.35 | [0.83,1.88] | 3.00 | [2.48,3.55] |
| $ß_{HOP}$ | 1.45 | [1.25,1.73] | 1.86 | [1.61,2.12] |
| $ß_2$ | 9.87 | [8.10,11.57] | 6.88 | [6.01,7.81] |
| ϕ | 0.19 | [0.10,0.29] | 0.14 | [0.07,0.20] |

**Table S2**. Proportion of correct S1 choice predictions by the winning model Q + TRIAL + HOP.

| Data Set | Min | 25th percentile | Median | Mean | 75th percentile | Max |
|---|---|---|---|---|---|---|
| data1 | .519 | .631 | .760 | .746 | .842 | .916 |
| data2 | .505 | .687 | .767 | .754 | .829 | .977 |

*Note.* Summary statistics are based on the comparison of individuals' choices with model predictions (i.e. simulated choices), which were averaged for each data set separately.

**Table S3.** *Results from model comparison of the best two variants in the full sample from Gillan and colleagues (2016) using leave-one-out cross-validation (LOO).*

| Data Set | Model | *-elpd diff* | *se diff* | *WAIC* |
|---|---|---|---|---|
| data2_full | Q + HOP | -98.3 | 20.6 | 35905.27 |
| (N=548) | Q + TRIAL + HOP | 0.0 | 0.0 | 35871.03 |

*Note*. The difference in the expected log pointwise predictive density (*elpd diff*) and standard error of the difference (*se diff*). These values show the results of a model comparison using LOO estimates. Each model is compared to the preferred model Q + TRIAL + HOP), as there is no difference between the winning model and itself, values in the first column are always zero.

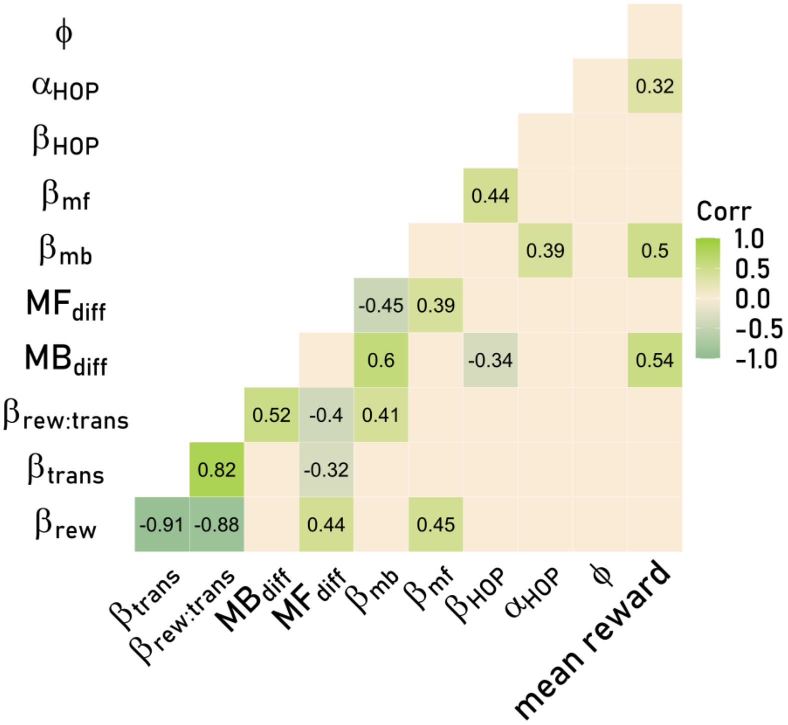


**Figure S3. Association of Model-Agnostic and Model-Derived Measures.** Individual model parameter estimates are based on model variant Q + TRIAL + HOP and data set data1. Numeric values depict the correlation coefficients, blank cells indicate non-significant associations. ß$_{rew}$,ß$_{trans}$, ß$_{rew:trans}$: regression weights for main effects of reward, transition type and their interaction; $MB_{diff}$, $MF_{diff}$: differences scores of MB and MF influences on S1 stay probabilities respectively; ß$_{MB}$,ß$_{MF}$: MB and MF S1 choice parameters from the winning model; ß$_{HOP}$: S1 higher order perseveration parameter; mean reward: mean reward gained throughout TST (300 trials).
